# MDC-MobileNetV3: A Lightweight Multi-Scale Hierarchical Attention Network for Remote Sensing Scene Classification

**DOI:** 10.3390/s26134174

**Published:** 2026-07-02

**Authors:** Haonan Liu, Xiao Wang, Jialong Sun, Xingchi Yang, Zhilong Wang

**Affiliations:** School of Marine Technology and Geomatics, Jiangsu Ocean University, Lianyungang 222005, China; 2024220220@jou.edu.cn (H.L.); 2025210256@jou.edu.cn (X.Y.); 2025210252@jou.edu.cn (Z.W.)

**Keywords:** MobileNetV3, MSFE, DFWF, remote sensing scene classification

## Abstract

Remote sensing scene classification remains challenging due to substantial object-scale variations, complex background interference, and high inter-class similarity. To address these issues, a lightweight classification framework, termed MDC-MobileNetV3, is proposed based on the MobileNetV3-Large backbone. The framework integrates a Multi-Scale Feature Extraction (MSFE) module for capturing spatial information at different receptive fields, a Dynamic Feature Weighted Fusion (DFWF) mechanism for adaptive feature recalibration, and the hierarchical CBAM attention strategy to enhance discriminative region representation. The model achieved high classification accuracies of 99.52%, 91.54%, 96.48%, 97.35%, 92.43%, and 99.72% on the UC Merced, WHU-RS19, NWPU-Resisc45, AID, CLRS, and PatternNet benchmark datasets, respectively, validating the effectiveness of the proposed framework, while maintaining a lightweight architecture with approximately 4.35 M parameters. In addition, Grad-CAM visualizations indicate that the model effectively focuses on semantically meaningful regions and suppresses irrelevant background information. The results confirm that the proposed framework provides a favorable trade-off between classification accuracy, model lightweight design, and model interpretability for remote sensing scene understanding.

## 1. Introduction

The continuous expansion of high-resolution remote sensing archives, together with the rapid evolution of deep neural architectures, has substantially broadened the applicability of Earth observation technologies in domains such as environmental monitoring [1], land-use analysis [2], urban management [3], and disaster assessment [4]. Within this context, remote sensing scene classification serves as a fundamental task that aims to infer semantic scene categories from aerial or satellite imagery, thereby providing essential information for large-scale geographic understanding and intelligent decision-support systems.

Despite considerable progress achieved over the past decade, reliable scene interpretation remains challenging. Numerous algorithms have been developed and extensively evaluated for remote sensing scene classification. From the perspective of traditional machine learning approaches such as Support Vector Machine (SVM) [5], Random Forests (RF) [6], and Extreme Gradient Boosting (XGBoost) [7] have been widely applied and optimized for remote sensing image data analysis. More recently, the rapid advancement of deep learning has further accelerated progress in this field. Representative architectures, including classic Convolutional Neural Network (CNN) [8], Feature Pyramid Network (FPN) [9], and most algorithms based on the Transformer architecture [10], have demonstrated remarkable performance in image classification, semantic segmentation, and object detection tasks. Nevertheless, despite these advances, remote sensing scene classification under complex geographic conditions remains a highly challenging problem with substantial room for further improvement in terms of feature representation, robustness, and generalization capability.

Building on the research into the aforementioned algorithms, recent years have witnessed extensive efforts by researchers to optimize and innovate upon existing various algorithms. In terms of improving and optimizing machine learning based algorithms, Palash et al. [11] proposed an SVM classifier based on principal component analysis (PCA) for linear unsupervised statistical change detection, demonstrating that effective feature extraction can significantly enhance the classification performance of original datasets. Tassi et al. [12] integrated the Simple Non-Iterative Clustering (SNIC) algorithm within the Google Earth Engine (GEE) platform for image segmentation, while employing the GLCM (Gray-Level Co-occurrence Matrix) to extract image texture features, and ultimately achieved effective classification performance using Random Forest (RF) and Support Vector Machine (SVM). Furthermore, Jiao et al. [13] proposed an APSO-CNN-XGBoost model, in which an improved adaptive particle swarm optimization (APSO) algorithm was utilized for hyperparameter optimization. By jointly optimizing the spatial feature extraction capability of Convolutional Neural Network (CNN) and the classification-oriented feature representation of XGBoost, the proposed method effectively mitigated the tendency of traditional PSO algorithms to become trapped in local optima, thereby improving the classification accuracy of remote sensing images.

To enhance the discriminative power of features, researchers have explored various strategies to optimize CNN architectures, including the refinement of loss functions and the incorporation of attention mechanisms. For example, Cheng et al. [14] proposed a discriminative CNN (D-CNN) model regularized by metric learning to address the common challenges in remote sensing scenes, namely large intra-class variability and small inter-class separability. By optimizing the discriminative objective, their method significantly improved the inter-class separability of features. From a feature fusion perspective, Chaib et al. [15] introduced Discriminant Correlation Analysis (DCA) to merge and reduce the dimensionality of the raw features extracted from VGG-Net, effectively alleviating the redundancy problem associated with high-dimensional representations. With the rise of attention mechanisms, Tong et al. [16] designed a dual channel-spatial attention module based on DenseNet, enabling the novel CAD network to adaptively focus on key feature regions and suppress interference from background noise. Furthermore, to better exploit the sequential nature of hyperspectral data, Hong et al. [17] pioneered the integration of Transformer architecture into remote sensing classification by proposing SpectralFormer, a novel backbone that employs cross-layer skip connections to overcome the limitations of conventional CNNs in modeling the sequential attributes of spectral features.

To address the challenge of significant scale variation and limited object sizes in remote sensing imagery, algorithm optimization has primarily focused on multi-scale feature aggregation and spatial structure modeling.

Xu et al. [18] proposed the Lie Group lightweight multi-scale network (LGLMNet). A dual-branch architecture is employed to first extract low-level features via Lie group machine learning and process high-level semantic information using parallel depthwise separable convolution modules, thereby achieving multi-scale perception. Finally, a spatial-channel collaborative attention mechanism is utilized to enable efficient global–local modeling. This approach effectively enhances scene representation capabilities while maintaining a lightweight structure. Yuan and Wang [9] proposed a Hierarchical Contextual Feature-Preserved Network (HCFPN) that combines the hierarchical feature extraction capabilities of CNNs with a Transformer-driven cross-layer attention mechanism; this approach aims to preserve hierarchical contextual information and enhance structural awareness, thereby improving scene discrimination in remote sensing imagery. Zheng et al. [19] proposed a Lightweight Dual-Branch Swin Transformer (LDBST) that utilizes a CNN branch to capture local spatial patterns, while a Swin Transformer branch modeled long-range contextual dependencies. By integrating Conv-MLP with a dual-branch feature learning strategy, the proposed framework effectively enhances scene classification capabilities while significantly reducing parameter overhead, highlighting the potential of lightweight hybrid architectures for remote sensing scene classification tasks.

Although these advances have improved classification performance, several limitations remain unresolved. Firstly, traditional conventional network (CNN)-based approaches predominantly rely heavily on fixed receptive fields, restricting their ability to capture discriminative patterns across diverse spatial scales. Second, direct multi-scale feature aggregation often introduces redundant channel responses, resulting in inefficient feature utilization and weakened semantic discriminative power. Third, attention mechanisms are frequently applied directly to backbone features, which may limit their effectiveness when feature representations still contain substantial redundancy and noise. Finally, many high-performing architectures, including VGG and ResNet variants, achieve superior accuracy at the cost of increased computational complexity, limiting their deployment on resource-constrained platforms such as drones and edge computing devices.

To address these limitations, this paper proposes an efficient hierarchical attention framework with collaborative feature enhancement: MDC-MobileNetV3. Designed for lightweight remote sensing scene classification, the main contributions are as follows:

1. A heterogeneous multi-scale feature extraction (MSFE) module is designed. This module integrates complementary receptive field generation strategies, rather than traditional parallel convolutions, enabling more comprehensive capture of local texture, expanded receptive field semantics, and pooling contextual information, avoiding the computational overhead of traditional pyramid architectures.
2. A dynamic feature weighted fusion (DFWF) module is proposed. This module adaptively recalibrates the heterogeneous contributions of multi-scale feature channels before feature refinement and suppresses redundant background information. Unlike traditional channel weighting, it explicitly alleviates the branch-wise feature imbalance problem caused during multi-scale fusion.
3. To distinguish traditional CBAM from direct application to the backbone network, an improved hierarchical CBAM module is designed. This module achieves complementary optimization of channel-aware semantic information and spatial discriminative target localization, thereby enhancing robustness to background noise and scale variations.
4. These three modules are jointly organized into a progressive feature enhancement framework, rather than being stacked independently, thus achieving an effective balance between representational power and lightweight deployment.

Furthermore, to comprehensively evaluate the proposed approach, experiments are conducted on six widely used benchmark datasets: UC Merced, WHU-RS19, NWPU-Resisc45, AID, CLRS, and PatternNet. These datasets encompass substantial variations in scene categories, object scales, and semantic complexity, providing a rigorous testing platform for assessing model generalization and representation capability. Experimental results demonstrate that the proposed framework achieves a good balance between classification accuracy, computational efficiency, and model interpretability, validating the effectiveness of the proposed design.

The remainder of this paper is organized as follows: Section 2 reviews relevant work on remote sensing scene classification; Section 3 details the proposed methods, including MSFE, DFWF, and the hierarchical CBAM modules; Section 4 describes the experimental setup and benchmark datasets; Section 5 presents and discusses the experimental results; and finally, Section 6 summarizes the entire paper and looks forward to future research directions.

## 2. Related Work

### 2.1. Evolution of Attention Mechanisms in CNNs

Convolutional Neural Networks (CNNs) achieve automatic extraction of high-level semantic features from raw pixels through multiple layers of convolution, pooling, non-linear activation functions, and fully connected layers, and have consequently become the fundamental framework for remote sensing scene classification. The fundamental principle of CNNs lies in the utilization of local receptive fields and parameter-sharing mechanisms, which enable effective modeling of spatial hierarchical structures while substantially reducing computational complexity.

Early CNN models primarily relied on relatively shallow architectures, enhancing feature representation capabilities by increasing the size of convolutional kernels or channel dimensions. Subsequently, network depth became a key direction for performance enhancement. By stacking multiple convolutional layers, deeper networks can learn more abstract and complex feature representations. However, this increased depth also introduces challenges such as vanishing gradients and optimization difficulties. To address these challenge, residual connections were introduced. By enabling direct information flow through shortcut paths, this mechanism substantially alleviates gradient propagation barriers and has become a standard component in modern deep CNN architectures [20].

While pursuing higher accuracy, the computational complexity and parameter counts of models have increased dramatically. To accommodate the resource constraints of mobile devices and real-time applications, lightweight CNN architectures have emerged. The most influential innovation is Depthwise Separable Convolution, which decouple the conventional convolution operations between channel and spatial dimensions, performing Depthwise convolution first, and then pointwise convolution. This design substantially reduces both parameter count and computational cost [21]. Building upon this foundation, inverted residual structures and linear bottlenecks were introduced to further improve feature propagation efficiency while preventing non-linear activations from destroying low-dimensional information [22]. More advanced lightweight designs combine Neural Architecture Search (NAS) with efficient activation functions such as hard-swish replacing Rectified Linear Unit (ReLU), along with channel attention mechanisms such as the Squeeze-and-Excitation (SE) module. by using global average pooling to compress channel information and employing the fully connected layers to generate channel weights. This module significantly improves the model’s ability to focus on important channels with minimal additional parameter overhead [23].

To further enhance the model’s perception to critical regions, attention mechanisms have been extended. Channel attention focuses on “what” are the most informative features, while spatial attention determines “where” the important information lies. The Convolutional Block Attention Module (CBAM) combines both approaches, first generating channel attentions and subsequently producing a spatial attention map, thereby achieving dual adaptive weighting across both channel and spatial dimensions. This composite attention mechanism effectively enhances the model’s ability to extract salient features in complex scenes without significantly increasing the computational burden, making it particularly suitable for remote sensing images characterized by high inter-class similarity and strong background interference [24].

The progressive development of CNN architectures—from deeper networks and lightweight designs to attention enhancement—has provided a robust technical foundation for constructing efficient and high-precision models for remote sensing scene classification. Building upon this foundation, the present study integrates the CBAM attention mechanism into a lightweight MobileNetV3 framework to achieve further optimization in both performance and efficiency.

### 2.2. Multi-Scale Feature Extraction

In remote sensing image scene classification tasks, due to substantial scale variations among ground objects, such as airports, farmlands, and building clusters, present significant challenges for feature representation. Feature extraction strategies relying on a single receptive field often struggle to capture both fine-grained local textures and global semantic context at the same time. Consequently, multi-scale feature extraction has become a critical approach for improving classification performance.

Zhang et al. (2019) [25] proposed a Multi-scale Deep Feature Representation (MDFR) framework, in which multi-level deep features were extracted from a pre-trained Convolutional Neural Network and subsequently filtered. Local features are then selected via a feature map selection algorithm and fused with global features. This strategy effectively captured discriminative information across different spatial scales while reducing feature redundancy, thereby improving the classification accuracy of remote sensing scenes. Dai et al. (2024) [26] proposed a multi-scale densely residual correlation network that incorporated a multi-stream feature extraction module to parallelly learn hierarchical representations at different scales. Through densely residual connected feature fusion, the proposed framework enhanced feature propagation and significantly improved scene discrimination capability under complex background conditions.

Wang et al. (2022) [27] developed a model based on Multi-Scale Residual Split Network (MSRes-SplitNet), in which the MSRes block to extracts multi-scale features. By integrating a split-attention module across multiple channels, the model aggregates global and local feature information through convolutional operations to form multi-scale representations, effectively alleviating the problem of few-shot learning. Lu et al. (2024) [28] further proposed the Multi-domain Semantic High-order Network (MSHNet), which embeds multi-scale and multi-resolution semantic modules within the wavelet domain. Leveraging Factorized Bilinear Coding (FBC) to capture rich statistical features and employing separable pooling blocks for structured multi-scale feature fusion, the method better preserves the spatial structural information of remote sensing imagery. Xia et al. (2022) [29] developed a Multi-scale Vision Transformer (MS-ViT) that simultaneously captures global and local information through a dedicated multi-scale feature learning and by validated strong robustness to scale variations in remote sensing scene classification.

These multi-scale feature extraction techniques provide a solid foundation for the further optimization of lightweight models. When combined with attention mechanisms, they enable more effective handling of the scale diversity that is prevalent in remote sensing imagery.

### 2.3. Hybrid Multi-Scale Attention Networks

Recent studies indicate that relying solely on a single attention mechanism or a multi-scale feature extraction strategy is insufficient to effectively address challenges such as scale variations and background interference in remote sensing scene classification. While attention mechanisms like SE, CBAM, and ECA successfully enhance discriminative feature responses, they typically operate on pre-extracted feature representations and lack explicit scale-awareness. Conversely, although multi-scale feature extraction strategies improve the perception of objects across different spatial scales, they are prone to introducing redundant information and struggle to adaptively highlight key feature regions. Consequently, hybrid multi-scale attention networks—which integrate multi-scale representation learning with attention-guided feature optimization—have garnered increasing attention as a means to enhance model capabilities in scene understanding.

From the early research on multi-scale hybrid spatial pyramid modules, Zhao and Du (2016) [30] proposed a multi-scale MCNN algorithm architecture that extracts high-level spatial features from multi-scale training datasets and reorganizes them into a pyramid structure of multi-scale spatial information, thereby effectively improving the model’s classification accuracy for objects at different scales. From the perspective of the hybrid attention networks, Hong et al. (2021) [17] developed SpectralFormer, a novel backbone network based on the ViT framework that effectively integrates global self-attention with local sequence attention. By employing Group-wise Spectral Embedding (GSE) to realize local spectral attention and Cross-layer Adaptive Fusion (CAF) to enhance cross-layer memory, the model significantly improves hyperspectral image classification performance, representing a significant innovation in adapting Transformers to the field of hyperspectral remote sensing.

Zhang et al. (2021) [31] proposed a multiscale attention network (MSA-Network) that integrates a multi-scale processing module with channel-position attention. The multi-scale module learns features across various depths and receptive fields, while the attention module captures global attention features at the channel level and local attention features at the pixel level; by automatically focusing on salient regions, the network effectively enhances remote sensing scene classification performance. Bi et al. (2021) [32] propose a Multi-scale Staking Attention Pooling (MS^2^AP). This framework combines multi-scale dilated convolution operators with residual channel-spatial attention mechanisms. By leveraging multiple receptive fields and attention-guided feature enhancement, it extracts key local semantic information from feature maps while suppressing background interference, thereby effectively improving remote sensing scene classification performance.

Despite the significant progress made by the aforementioned hybrid multi-scale attention networks, several limitations remain to be addressed. Existing methods either rely on computationally expensive Transformer architectures or employ fixed feature aggregation strategies that fail to account for the varying importance of features across different scales. Furthermore, most attention mechanisms are applied to representations after feature aggregation rather than guiding the fusion process itself; consequently, achieving a balance between classification accuracy and computational efficiency through efficient multi-scale representation learning, adaptive feature weighting, and lightweight deployment remains a pressing challenge.

To address the aforementioned issues, this paper proposes the MDC-MobileNetV3 framework, which integrates a lightweight MobileNetV3 backbone network, multi-scale feature extraction (MSFE), dynamic feature-weighted fusion (DFWF), and a hierarchical CBAM-based feature optimization mechanism.

## 3. Method

### 3.1. Overall Architecture

To address the issues of significant scale variations, complex background interference, and subtle inter-class discrepancies in remote sensing images, a lightweight optical image classification framework, termed MDC-MobileNetV3, is proposed in this study. The proposed architecture integrates three core modules: Multi-Scale Feature Extraction (MSFE), Dynamic Feature Weighted Fusion (DFWF), and the Hierarchical Convolutional Block Attention Module (CBAM). Through the synergistic coupling of these components, the framework achieves enhanced multi-scale representation capability and discriminative feature learning. The overall framework structure is shown as in Equation (1):

(1) $$F_{out}=H_{cls}\left(A_{CBAM}\left(F_{DFWF}\left(F_{MSFE}\left(B_{MNV3}\left(X\right)\right)\right)\right)\right)$$

**Figure 1:**
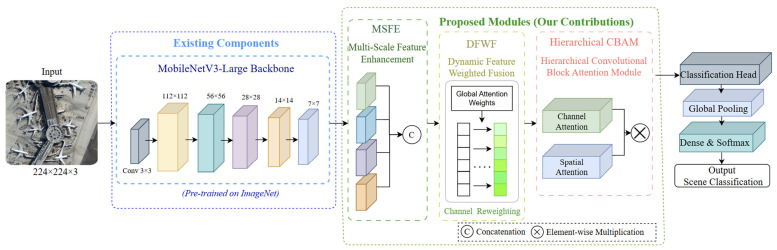
Model General Structure Diagram.

In the equation, X denotes the input image; B_MNV3_ denotes the MobileNetV3 backbone network; F_MSFE_ corresponds to the Multi-Scale Feature Extraction module; F_DFWF_ denotes the Dynamic Feature Weighted Fusion module; A_CBAM_ represents the attention mechanism based on the hierarchical Convolutional Block Attention Module; and H_cls_ denotes the classification head. The overall network architecture and processing flow are illustrated in Figure 1.

**Figure 2:**
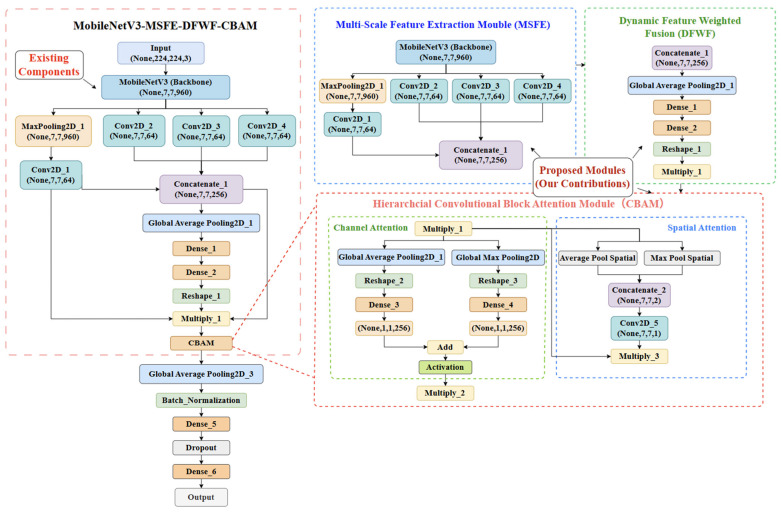
Model Detailed Structure Flowchart.

The input image X is first processed by the pre-trained MobileNetV3-Large backbone to extract high-level semantic features. A multi-branch convolutional structure is then employed for multi-scale feature extraction. Adaptive feature fusion is subsequently realized through the dynamic weighting mechanism. Finally, the hierarchical CBAM attention module is introduced to strengthen responses in critical regions, with the final prediction generated by the classification head, as shown in the model structure flowchart (Figure 2). While maintaining high computational efficiency and a lightweight design, the proposed model substantially enhances feature representation capability and classification robustness. During training, EarlyStopping and ModelCheckpoint callbacks are utilized, and model performance is systematically evaluated through classification reports, confusion matrices, and Grad-CAM analysis.

### 3.2. Backbone Network

**Figure 3:**
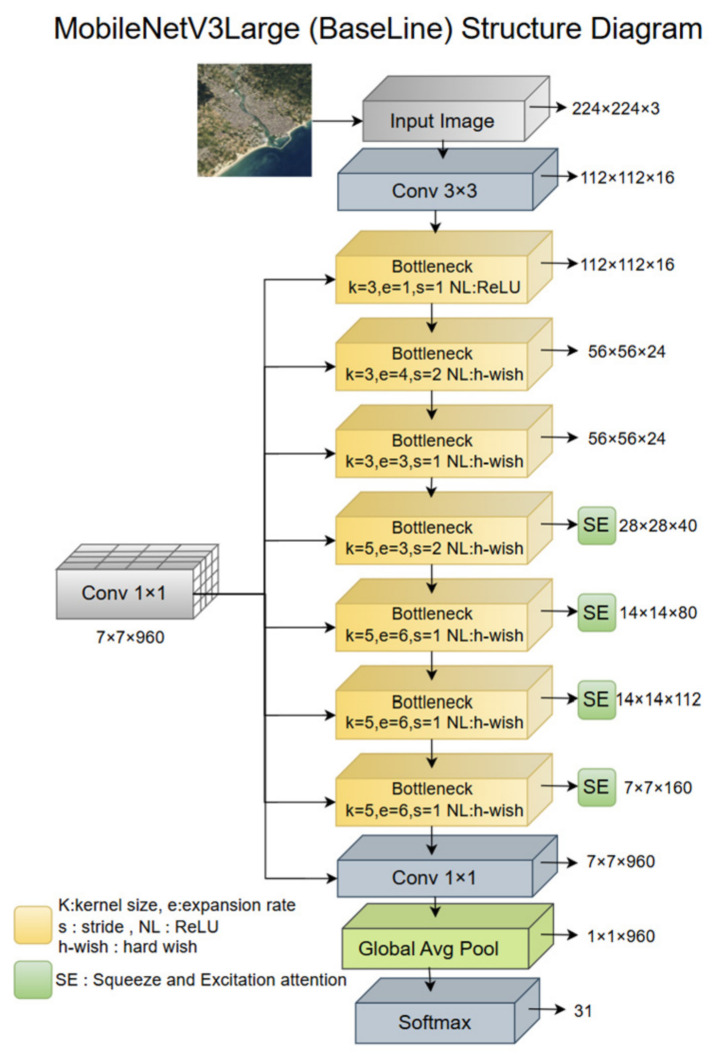
MobileNetV3 Baseline Structure Diagram.

To achieve efficient feature extraction while maintaining a lightweight design, this paper adopts MobileNetV3-Large as the backbone network for basic feature extraction, with its overall architecture illustrated in Figure 3. This network was proposed by Howard et al. (2019) [23] for Neural Architecture Search (NAS) design and achieves an excellent balance between computational efficiency and classification performance.

As shown in Figure 3, MobileNetV3-Large employs a standard 3 × 3 convolution layer as its input, mapping images of size 224 × 224 × 3 into the feature space. Subsequently, the network is composed of multiple cascaded inverted residual bottleneck structures, comprising modules such as depth-separable convolutions, point-wise convolutions, non-linear activation functions, and SE attention modules. As the network depth increases, the spatial resolution of feature maps gradually decreases from 112 × 112 to 7 × 7, while the number of channels is expanded to 960, thereby enabling hierarchical representation learning from low-level texture features to high-level semantic features. Finally, global average pooling is applied to generate a 1 × 1 × 960 global feature vector, which serves as a high-quality input for subsequent modules.

To further reduce computational complexity, MobileNetV3 adopts the depthwise separable convolution structure [21], whose computational cost can be expressed as Equation (2):

(2) $$Cost=D_{k}^{2}\cdot M\cdot H\cdot W+M\cdot N\cdot H\cdot W$$

In the equation, D_k_ denotes the kernel size, M and N denote the numbers of input and output channels, respectively, and H and W indicate the spatial dimensions of the feature map. In comparison with standard convolution, as expressed in Equation (3):

(3) $${Cost}_{standard}=D_{k}^{2}\cdot M\cdot N\cdot H\cdot W$$

This type of convolution significantly reduces parameter scale and computational overhead, enabling the model to achieve strong lightweight characteristics while preserving high performance. To further enhance feature representation capability, MobileNetV3 incorporates the Squeeze-and-Excitation (SE) module [33] into selected bottleneck structures. The core idea of the SE module is to perform adaptive recalibration of features through a channel attention mechanism. Specifically, the Squeeze operation is formulated as Equation (4):

(4) $$z_{c}=\frac{1}{H\times W}\sum_{i=1}^{H}\sum_{j=1}^{W}u_{c}\left(i,j\right),\;\;\;\;\;c=1,2,\ldots C$$

In the equation, H × W represents the spatial dimensions of the feature map, C denotes the total number of channels, and z ∈ R^c^ represents the channel descriptor vector. Channel weights are then generated via the Excitation layer (two fully connected layers) to achieve adaptive feature enhancement and suppression of redundant features.

In addition, MobileNetV3 introduces the hard Swish (h-swish) activation function [23] as shown in Equation (5):

(5) $$h_{Swish}\left(x\right)=x\cdot \frac{Relu6\left(x+3\right)}{6}$$

In the equation, x represents the input feature. The ReLU6 activation function is a clipped nonlinearity with an output range of [0, 6]. This formulation maintains non-linear representational capacity while enhancing computational efficiency. By initializing with ImageNet pre-trained weights, the model demonstrates strong feature transfer capabilities, providing high-quality semantic features for subsequent modules. Finally, the 7 × 7 × 960 output feature dimension generated by the backbone network offers sufficient and complementary semantic information for downstream modules, effectively avoiding the representational bottlenecks that may arise from performing complex fusion directly on shallow-layer features.

### 3.3. Multi-Scale Feature Extraction (MSFE) Module

**Figure 4:**
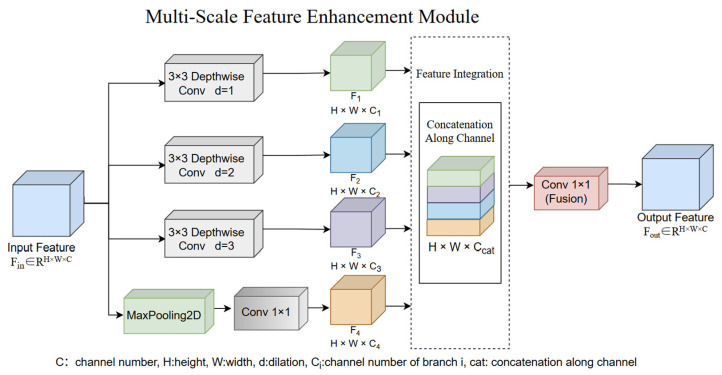
MSFE Module Structure Diagram.

Building upon the 7 × 7 × 960 high-level semantic features output by the MobileNetV3-Large backbone network, we propose a lightweight Multi-Scale Feature Extraction (MSFE) module to effectively address the substantial scale variation of ground objects in remote sensing images. The overall structure of the MSFE module is illustrated in Figure 4.

As shown in Figure 4, the module comprises four parallel convolutional branches that extract distinct features from the same input feature map:

(6) $$F_{i}={Conv}_{i}\left(F\right),\;\;\;\;i\in \left\{1,2,3,4\right\}$$

In the equation, F ∈ R^H×W×C^ represents the input feature map; Conv_1_ represents a 3 × 3 convolution for channel compression, Conv_2_ is a 3 × 3 convolution for extracting local features, Conv_3_ employs a large-kernel convolution to expand the receptive field, and Conv_4_ is a pooling branch for capturing global context.

The three convolutional layers (Conv_1_–Conv_3_) operate directly on the backbone feature map, each producing 64-channel features. Additionally, a max-pooling (MaxPooling2D) branch followed by a 1 × 1 convolution generates the fourth 64-channel feature. Finally, the four features are concatenated along the channel dimension via a Concatenate operation to form a 7 × 7 × 256 multi-scale feature, as shown in Equation (7):

(7) $$F_{MSFE}={⊕}_{i=1}^{4}F_{i}$$

In the formula, ⊕ denotes the concatenation operation along the channel dimension, and F_MSFE_ ∈ R^H×W×C’^,C’ refers to the number of channels after concatenation.

The motivation for designing this module stems from the pronounced scale variations of ground objects in remote sensing imagery. For example, small-scale airports resemble large airport runways in terms of local structure, making it difficult for a single receptive field to capture both local texture and global semantic information simultaneously. Furthermore, traditional single-scale CNNs are susceptible to losing critical information from small targets or failing to suppress background noise. In contrast, the proposed MSFE module structure designed in this paper is capable of simultaneously capturing local details, mid-scale structures, and global semantic information. By explicitly modeling multiple receptive fields through parallel convolutions and pooling operations, it achieves hierarchical feature extraction. Drawing inspiration from the Feature Pyramid Network (FPN) [34], the module adopts lightweight convolutions and pooling strategies to avoid information loss and excessive computational overhead typically associated with up- and down-sampling operations [35]. Moreover, the designed MSFE module is inherently compatible with the depth-separable convolutional structure of MobileNetV3, significantly enhancing feature representation capabilities while maintaining the model’s lightweight nature. This design substantially improves feature discriminability and model robustness, providing a richer and more complementary feature foundation for subsequent modules and effectively mitigating performance degradation arising from scale variations.

### 3.4. Dynamic Feature Weighted Fusion (DFWF) Module

**Figure 5:**
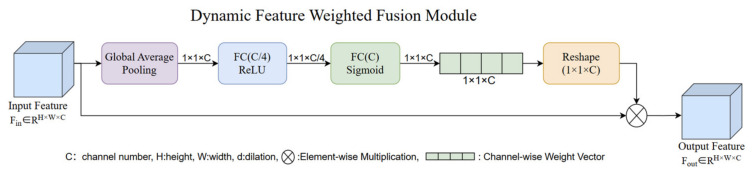
DFWF Module Structure Diagram.

Building upon the 7 × 7 × 256 fused feature representations generated by the Multi-Scale Feature Extraction (MSFE) module, a Dynamic Feature Weighted Fusion (DFWF) module is further proposed to alleviate the imbalance of channel contributions among features of different scales. The structure of the proposed DFWF module is illustrated in Figure 5.

The proposed module first employs Global Average Pooling (GlobalAveragePooling2D) to compress spatial information into compact channel descriptors. Subsequently, two fully connected layers (Dense_64_ → Dense_256_) are utilized to learn channel attention weights. These weights are then reapplied to the original fused feature map through Reshape and Multiply operations, thereby achieving adaptive recalibration along the channel dimension.

The motivation for this module lies in the fact that, following concatenation in the Multi-Scale Feature Extraction (MSFE) module, the relative importance of contributions from different branch channels varies. Some channels may carry redundant background information, while others carry critical discriminative semantics. Traditional approaches typically adopt equal-weight concatenation for feature fusion, as shown in Equation (8):

(8) $$F=\sum_{i}F_{i}$$

Such fusion strategies neglect the heterogeneous importance of feature channels and are prone to introducing redundant information. Therefore, to address this limitation, the proposed Dynamic Feature Weighted Fusion (DFWF) module performs spatial compression on the input feature representations through global average pooling, transforming two-dimensional feature maps into compact channel description vectors, as expressed in Equation (9):

(9) $$z=GAP\left(F_{MSFE}\right)$$

In the equation, GAP(·) represents the global average pooling operation that squeezes the feature map into a channel description vector z ∈ R^C’^. Subsequently, channel-wise attention weights are learned via two fully connected layers, as expressed in Equation (10):

(10) $$w=\sigma \left(W_{2}\cdot \delta \left(W_{1}z\right)\right)$$

$W_{1}\in R^{\frac{c\prime }{r}\times C\prime }$ and $W_{2}\in R^{C\prime \times \frac{c\prime }{r}}$ denote learnable weight matrices, in which r represents the channel reduction ratio, $\delta \left(\;\right)$ corresponds to the Rectified Linear Unit activation function, and σ( ) denotes the Sigmoid activation function. The resulting vector $w\in R^{C\prime }$ represents the learned channel-wise attention weights. Feature recalibration is subsequently performed according to Equation (11):

(11) $$F_{DFWF}=F_{MSFE}⊙w$$

In the equation, ⊙ denotes channel-wise broadcast multiplication, which is be employed to achieve weighted rescaling of feature maps.

The DFWF module is essentially a lightweight channel attention rescaling mechanism. Its core lies in adaptively assigning channel weights through global context modeling, thereby achieving effective feature selection and suppression. The design draws inspiration from the Squeeze-and-Excitation (SE) module for single-scale features [36], but the DFWF module is more suited to multi-scale feature fusion. By dynamically generating channel weights, it enables adaptive selection of informative features and effectively alleviates the channel imbalance problem inherent in multi-scale feature fusion (MSFE) [37]. In addition, while the DFWF module emphasizes global channel dimension, the Hierarchical CBAM mechanism further refines features in the spatial dimension. The two form a complementary relationship and can also provide cleaner input features for Hierarchical CBAM module [38]. Compared with static fusion strategies, the DFWF module enables the model to focus more effectively on discriminative multi-scale cues, improving decision boundaries and significantly enhancing the model’s representational power and discriminability.

### 3.5. Hierarchical Convolutional Block Attention Mechanism (CBAM) Module

**Figure 6:**
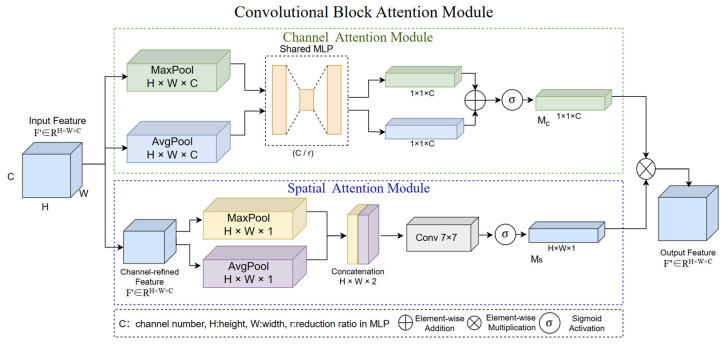
Hierarchical CBAM Module Structure Diagram.

To further suppress background interference in complex remote sensing scenes and enhance the responses of discriminative regions, the Hierarchical Convolutional Block Attention Module (CBAM) is introduced after the Dynamic Feature Weighted Fusion (DFWF) module, and its overall architecture is illustrated in Figure 6. Hierarchical CBAM adopts a sequential attention modeling strategy composed of channel attention and spatial attention [39], enabling adaptive refinement of feature representations from the perspectives of channel importance and spatial saliency, respectively, thereby facilitating more discriminative feature learning.

In contrast to conventional applications that apply CBAM directly to backbone network, this paper embeds the CBAM module after the Multi-Scale Feature Extraction (MSFE) and Dynamic Feature Weighted Fusion (DFWF) modules, enabling it to more fully represent the fused feature space. Specifically, the DFWF module first performs global channel recalibration on the multi-scale features to achieve coarse-grained feature selection. Subsequently, Hierarchical CBAM applies its sequential channel and spatial attention mechanisms for fine-grained refinement, forming a hierarchical attention mechanism. Finally, the serial structure computes global average pooling and global max pooling respectively, yielding two 7 × 7 × 1 descriptor maps. These representations are concatenated into a 7 × 7 × 2 feature map and subsequently processed by a 7 × 7 convolution layer to generate the spatial attention weight map, and spatial feature focusing is achieved via a Multiply operation.

Referring to Figure 6, for input features F ∈ R^H×W×C^, the channel attention is defined as follows:

(12) $$M_{c}\left(F\right)=\sigma \left(MLP\left(AvgPool\left(F\right)\right)+MLP\left(MaxPool\left(F\right)\right)\right)$$

In this equation, AvgPool( )and MaxPool( ) denote the global average and global max pooling operations, respectively, while MLP( ) denotes a weight-shared multi-layer connected network. Additionally, M_c_(F) ∈ R^1×1×C^ represents the channel attention weights, thereby yielding the channel-weighted output expressed as:

(13) $$F^{\prime }=M_{c}\left(F\right)⊗F$$

The spatial attention mechanism is defined as Equation (14):

(14) $$M_{s}\left(F^{\prime }\right)=\sigma \left(f^{7\times 7}\left(\left[Avg\left(F^{\prime }\right);Max\left(F^{\prime }\right)\right]\right)\right)$$

Avg( ) and Max( ) represent average pooling and max pooling along the channel dimension; represents channel-wise concatenation, while $f^{7\times 7}$ corresponds to a convolution operation with a kernel size of 7 × 7. With $M_{s}\left(F\prime \right)\in R^{H\times W\times 1}$ representing the spatial attention weights, the consequent output is defined as shown in Equation (15):

(15) $$F^{\prime \prime }=M_{s}\left(F^{\prime }\right)⊗F^{\prime }$$

$F^{\prime \prime }$ represents the feature output after weighting by spatial dimensions; combined with the joint integrating of channel and spatial attention mechanisms, the overall output is expressed as Equation (16):

(16) $$F_{CBAM}=M_{s}\left(M_{c}\left(F\right)\right)⨂F$$

The architectural motivation for this module stems from the inherent complexity of remote sensing images, typically characterized by cluttered backgrounds (such as vegetation, roads, and water bodies) and high inter-class similarities that render models susceptible to interference from irrelevant regions [40]. Traditional CNNs treat spatial locations and channel responses with equal weight during feature learning, which frequently leads to dispersed attention and inadequate emphasis on critical areas. The proposed Hierarchical Convolutional Block Attention Module (CBAM) addresses these limitations through a two-stage “channel-to-spatial” attention strategy, which separately models the complementary problems of identifying informative feature channels and discriminative spatial regions.

This design is lightweight and computationally efficient, introducing only a minimal number of additional param, which makes it highly suitable for lightweight network architectures. It avoids the substantial computational overhead of global attention mechanisms such as Transformers [41] while compensating for the limited global dependency modeling inherent in MobileNetV3’s local convolutions.

The module markedly improves the model’s focus on foreground targets and effectively mitigates the influence of background noise on classification decisions. Compared with the original CBAM, the improved strategy proposed herein performs attention allocation on higher-quality feature representations, thereby reducing redundant information interference, enhancing attention localization precision, and strengthening focus on key target regions. Relative to single SE or GAM modules [42], Hierarchical CBAM’s dual attention mechanism provides more comprehensive feature refinement and constitutes a key innovation for achieving high performance within a lightweight backbone.

## 4. Dataset and Experiment Setup

### 4.1. Dataset Description

**Figure 7:**
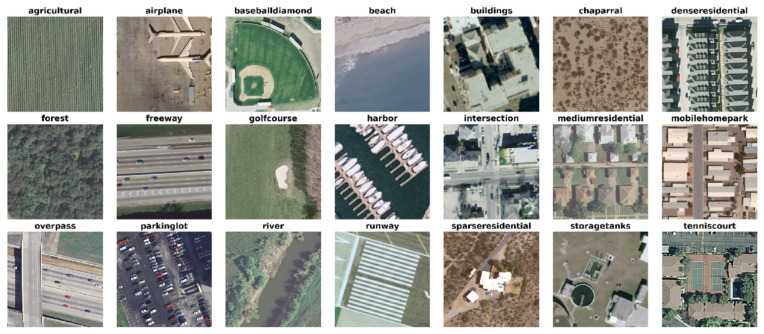
Representative Scene Samples from the UC Merced Land Use Dataset.

UC Merced Land Use Dataset: The UC Merced Land Use Dataset is one of the earliest established benchmarks for remote sensing scene classification. Released by the University of California, Merced, it contains 21 typical land-use scenes, with 100 images for each category, totaling 2100 images. All images are uniformly sized at 256 × 256 with a spatial resolution of approximately 0.3 m [43], as shown in Figure 7.

**Figure 8:**
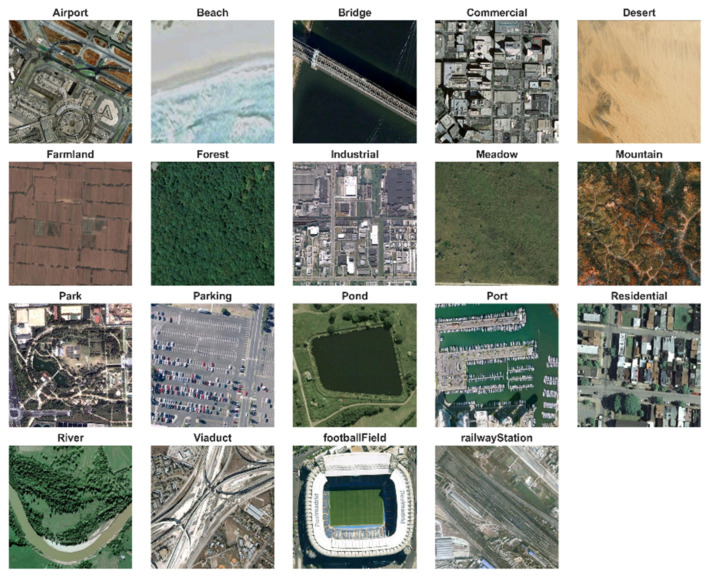
Representative Scene Samples from WHU-RS19 Dataset.

WHU-RS19: The WHU-RS19 Dataset, released by Wuhan University, is a medium-scale remote sensing scene classification benchmark. It contains 19 scene categories, a total of 1005 images, comprising approximately 50–55 samples per class. The image size is 600 × 600 pixels, which has a relatively high spatial resolution [44]. WHU-RS19 exhibits more complex spatial structures and texture distributions, thereby imposing higher requirements on feature extraction capability. Representative examples are shown in Figure 8.

**Figure 9:**
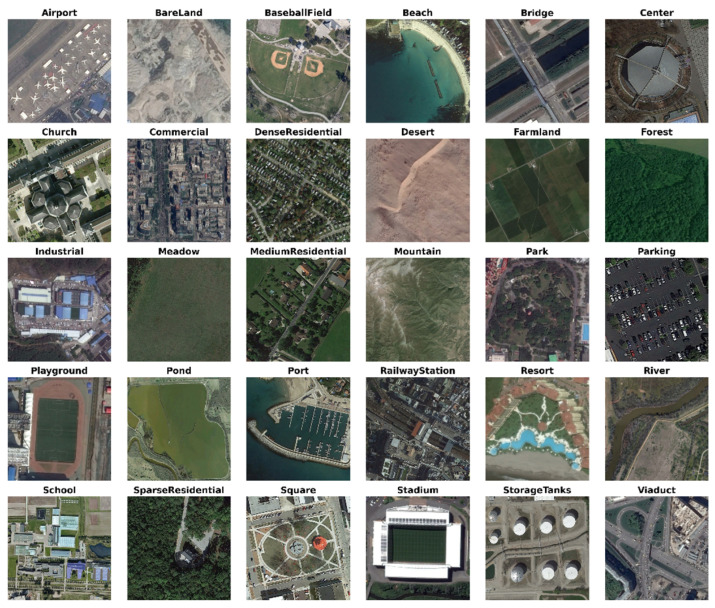
Representative Scene Samples from AID Dataset.

AID: The Aerial Image Dataset is a large-scale, high-resolution benchmark remote sensing image and one of the most widely adopted remote sensing scene classification datasets. Released by the Chinese Academy of Sciences, it comprises 30 scene classes, totaling 10,000 images. All images are sized 600 × 600 pixels, with spatial resolutions ranging from 0.5 m to 8 m [45], as shown in Figure 9.

**Figure 10:**
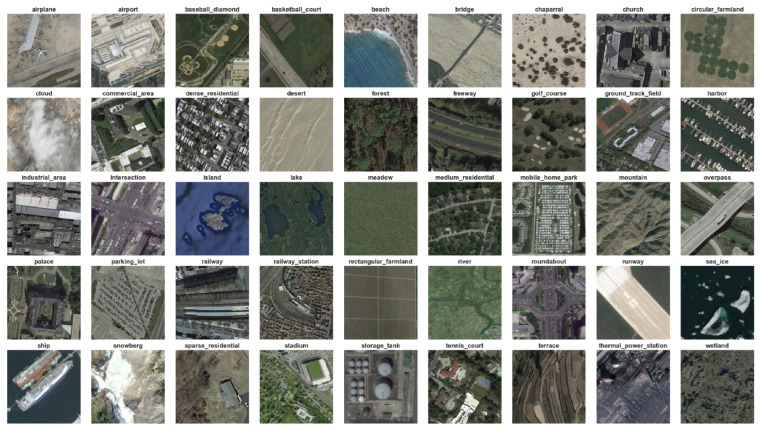
Representative Scene Samples from NWPU-Resisc45 Dataset.

NWPU-Resisc45: The NWPU-Resisc45 Dataset is one of the most widely used large-scale remote sensing scene classification datasets to date, released by Northwestern Polytechnical University. The dataset comprises 45 scene classes, each contain 700 images, totaling 31,500 images, and spatial resolutions ranging from 0.2 m to 30 m [8], as shown in Figure 10.

**Figure 11:**
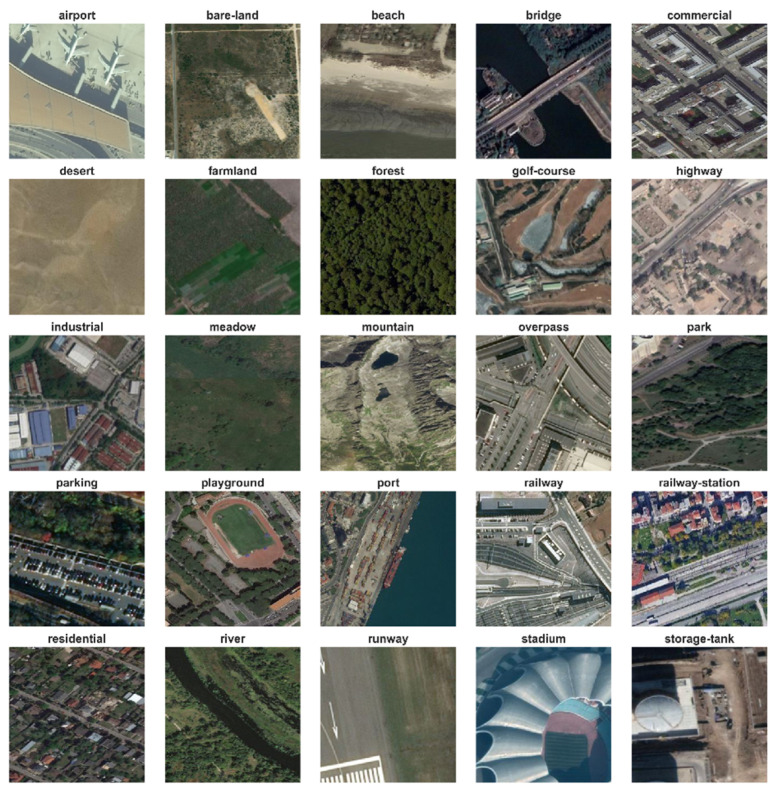
Representative Scene Samples from CLRS Dataset.

CLRS: The CLRS Dataset is one of the remote sensing scene classification datasets proposed in recent years, primarily derived from high-resolution remote sensing imagery of regions in China. It includes 25 scene categories, such as airports, bare ground, and beaches. There are 600 images per category, total 15,000 remote sensing images. All image dimensions are 256 × 256, with a spatial resolution ranging from 0.26 m [46], as shown in Figure 11.

**Figure 12:**
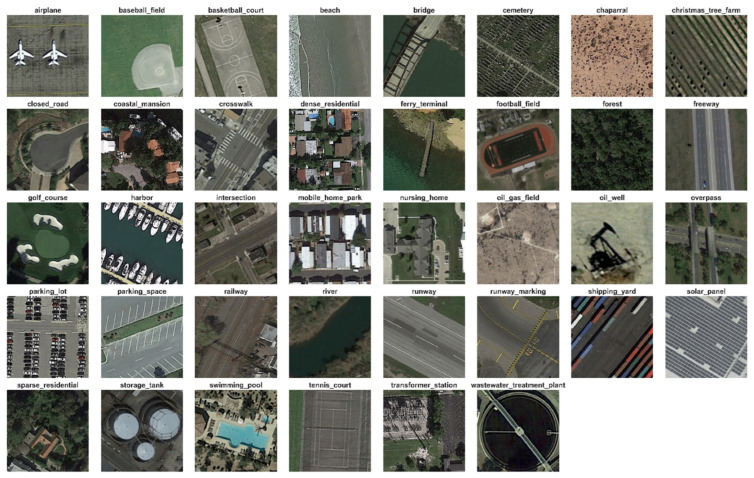
Representative Scene Samples from PatternNet Dataset.

PatternNet: The PatternNet Dataset is a high-quality, large-scale remote sensing image benchmark comprising 38 scene classes, with 800 images per class, total 30,400 images. All images are sized 256 × 256 pixels and feature high data quality with accurate annotations [47], as shown in Figure 12.

To provide a more intuitive comparison of the characteristics of the above datasets, their key attributes are summarized in Table 1.

### 4.2. Experimental Details

To validate the effectiveness of the approach proposed in this paper, all experiments were implemented using TensorFlow version 2.10.0 and the Keras deep learning framework. Model training and evaluation were conducted on a hardware platform accelerated by an NVIDIA RTX 4060 GPU to enhance model training efficiency.

1. Data Preprocessing:

Prior to model training, the input images underwent uniform preprocessing. All images were resized to a standardized dimension of 224 × 224 × 3 pixels, with their pixel intensities normalized to the [0, 1] range. In addition, data loading was performed using an ImageDataGenerator, and random shuffling was applied during training to enhance the generalization capability of the network.

2. Dataset Partitioning:

In this paper, a training-to-test ratio of 80% is adopted, with the training and test sets split randomly. To guarantee the reproducibility of the experimental outcomes, the random seed was fixed during the splitting process. To rigorously validate the experimental performance advantages of the proposed model against state-of-the-art (SOTA) algorithms under a fair baseline, a consistent training-to-test ratio was employed across the six different datasets used in this study.

3. Data Augmentation Settings:

To increase the diversity of training samples and mitigate overfitting, data augmentation was applied to the training set. This included random rotation within a range of ±30°, translation in both horizontal and vertical directions by up to 20% of the image dimensions, shearing with a shear factor of 0.2, scaling within a range of ±20°, and random horizontal flipping. The test set underwent only standardization and normalization; this ensured that performance evaluation was conducted on unaltered samples while preserving the generalization capabilities gained through data augmentation during training.

4. Model Training Settings:

During the training stage, the Adam optimizer [48] was employed to optimize network parameters. To facilitate stable convergence and improve optimization efficiency, a Cosine Decay learning-rate schedule [49] was employed during training. The learning rate was initialized at 1 × 10^−3^ and gradually decreased following a cosine function until it reached 1 × 10^−5^ at the end of training, while the categorical cross-entropy loss function was selected to accommodate multi-class remote sensing classification tasks. The batch size was set to 32 and the number of training iterations was set to 50, with parameter updates executed iteratively via backpropagation algorithm. To mitigate overfitting, a Dropout mechanism was incorporated within the fully connected layers. In addition, Batch Normalization was applied to normalize the feature, thereby enhancing the stability of the model and accelerating convergence.

5. Evaluation Metrics:

To comprehensively assess the model’s performance, classification accuracy (Accuracy) was adopted as the primary evaluation metric. In addition, the number of parameters (Params) and floating-point operations (FLOPs) were introduced as auxiliary metrics to evaluate the proposed framework from the perspectives of model scale and computational cost.

## 5. Results and Analysis

In this section, this paper provides a detailed description and discussion of the experimental results across six datasets. To rigorously evaluate the effectiveness of the proposed method, we compared it against a range of state-of-the-art models published in recent years, including ViT-B16 [50], EfficientNetB0, EfficientNetB1, EfficientNetB2, EfficientNetB3 [51], ConvNext [52], ShuffleNetV2 [53], NASNet [54], MobileNetV2 [22], MobileNetV4 [55], DenseNet121 [56], ResNet50 [20], ResNet50V2 [57], VGG16, and VGG19 [58]. All comparative experiments were conducted under consistent local deployment conditions. In addition, extensive ablation studies were performed to quantify the contribution of each proposed module.

### 5.1. Comparative Experiment

To thoroughly evaluate the effectiveness of the proposed method, Table 2 presents a comparative analysis of the MDC-MobileNetV3 model against 11 SOTA models on the AID dataset. To ensure fairness in the comparative experiments and consistency in the evaluation protocol, all models were trained and evaluated using identical training/test splits, data augmentation strategies, and optimizer settings, with comprehensive assessments based on accuracy, parameter count (Params), and FLOPS. Most comparative models utilized backbone networks with official ImageNet pre-trained weights obtained via the TensorFlow/Keras framework; however, models lacking compatible pre-trained weights—such as ConvNext, MobileNetV4, ShuffleNetV2, and the proposed MDC-MobileNetV3—were implemented independently and trained directly on the target dataset. Additionally, to optimize performance for EfficientNet-B1 and EfficientNet-B2, the input resolution was adjusted from 224 × 224 to their respective compatible sizes of 240 × 240 and 260 × 260. Despite minor differences in initialization strategies, the use of identical training conditions ensured consistency across the evaluation process for all models.

The experimental results demonstrate that the proposed MDC-MobileNetV3 model achieves superior classification performance on the AID dataset. Although the proposed framework remains slightly less competitive in terms of parameter scale compared with several classical lightweight architectures, including ShuffleNetV2, MobileNetV2, MobileNetV3, and MobileNetV4, ShuffleNetV2 exhibits the highest inference efficiency among the compared lightweight models. Meanwhile, other models also performed well in terms of FLOPs computation. In particular, the VIT-B16 model reaches 35.228 G FLOPs, indicating substantial computational complexity; however, its classification accuracy achieves only 88.85%, which is relatively low compared to other models.

In contrast, the proposed model achieves an overall classification accuracy of 97.35%, which is significantly higher than that of other models. Despite requiring only 0.57 GFLOPs, the proposed network maintains favorable inference efficiency and reduced computational overhead. These results indicate that the proposed architecture maintains a low computational footprint while simultaneously enhancing recognition precision within a lightweight framework, thereby providing compelling empirical evidence for the efficacy of the innovative modules introduced in this work.

**Figure 13:**
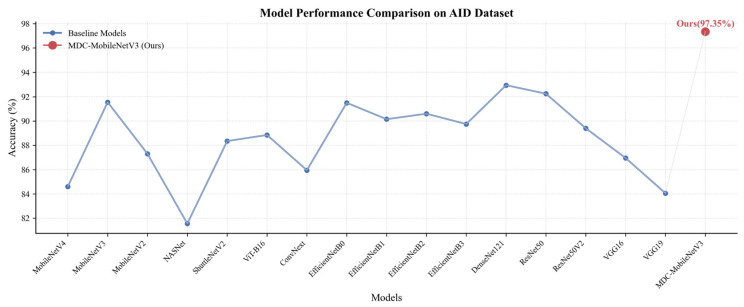
Line Chart of Accuracy for Each Model.

Meanwhile, this paper also conducts experimental comparisons with traditional models on the AID dataset. Combined with Table 3 and Figure 13 below, it can be found that although the proposed method is not as good as the classic CNN model in terms of FLOPs computation, it is far superior to the traditional method in terms of lightweight, accuracy, and computation speed.

**Figure 14:**
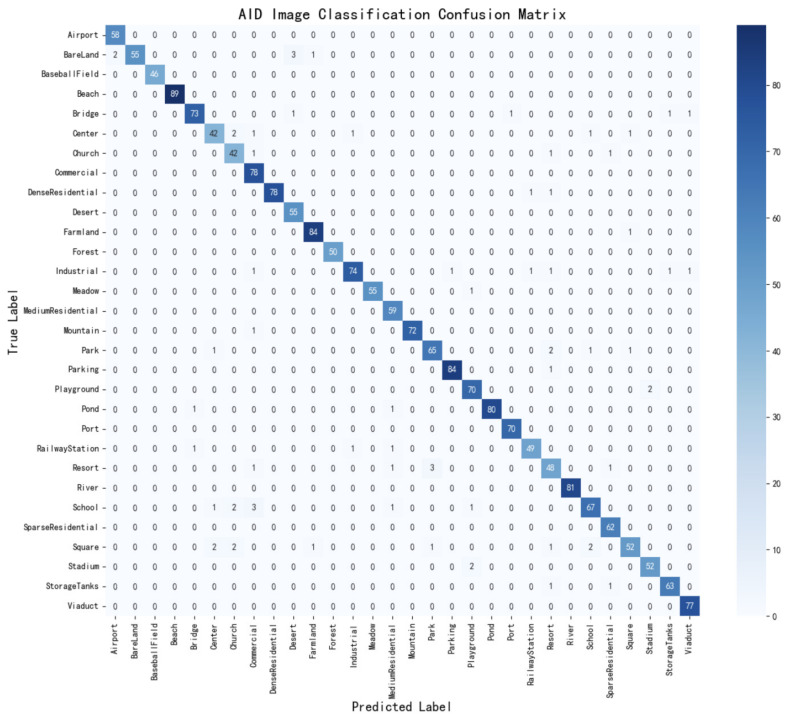
Confusion matrix of MDC-MobileNetV3 on the AID dataset.

To further evaluate the classification capability of MDC-MobileNetV3 in complex remote sensing scenes, this paper also conducts quantitative and qualitative analyses by combining classification reports with confusion matrices. As shown in Table 4, the model achieves high recognition accuracy across the majority of categories. Overall, the Precision, Recall, and F1-score metrics remain consistently high for most categories, with the majority exceeding 0.95. Meanwhile, the confusion matrix shown in Figure 14 exhibits a significant diagonal concentration distribution, indicating the proposed model’s good classification and discrimination capabilities on the AID dataset. Most scene categories are correctly classified, demonstrating the model’s excellent generalization ability and classification stability.

From the perspective of specific types and categories, categories with significant spatial structure and texture features (such as BaseballField, Beach, Forest, and River) have all reached saturation in all indicators. These scene categories generally contain clear geometric configurations or unique texture distributions, which are highly beneficial for effective modeling within the proposed (MSFE) module.

More specifically, the combined observations from Table 4 and Figure 14 indicate that the categories Church (0.875) and Resort (0.857) exhibit Precision values below 0.9, while Center (0.875) and Resort (0.889) present Recall values below 0.9. Similarly, the F1-score values below 0.9 for Center (0.894), Square (0.897), and Resort (0.873) remain comparatively lower than those of other categories. Furthermore, several representative confusion phenomena can be observed in the confusion matrix, including BareLand being misclassified as Desert, School as Commercial, and Resort as Park. These misclassification patterns are primarily attributed to the high spatial structural similarity among certain scene categories in remote sensing imagery, such as comparable building density distributions, limited texture discrepancies, and substantial background interference. Consequently, despite the incorporation of multi-scale feature modeling and attention mechanisms, a limited degree of category confusion remains unavoidable, but has been a marked improvement compared to traditional models. Importantly, the confusion matrix does not exhibit evident overfitting toward any specific category, indicating balanced classification capability across different scene classes.

The above results further demonstrate that the introduced MSFE module, DFWF mechanism, and Hierarchical CBAM attention mechanism effectively enhance feature representation capability. On the one hand, the multi-scale structure improves the model’s ability to perceive targets at different scales; on the other hand, the attention mechanism enhances the response of key regions and suppresses background interference, thereby reducing inter-class confusion.

### 5.2. Ablation Experiment

To systematically verify the effectiveness and synergistic effects of the proposed key modules, this paper conducts ablation experiments on the PatternNet dataset under identical experimental conditions, examining the multi-scale feature extraction module (MSFE), dynamic feature fusion module (DFWF), and the hierarchical CBAM attention mechanism introduced in the model. All experiments are based on the same data partitioning, training strategy and evaluation metrics to ensure the fairness and comparability of the results. To achieve a more precise evaluation, in addition to classification accuracy, Params and FLOPs, this paper introduces statistical metrics—specifically Mean Accuracy ± Standard Deviation (MA ± Std) calculated from multiple runs—as well as hardware efficiency metrics, including inference time, frames per second (FPS), CPU memory usage, and peak GPU memory consumption.

Experimental Setup: MobileNetV3-Large is used as the baseline model. Various improvement modules are progressively introduced on top of this to construct the comparison models listed in Table 5 below.

As can be observed from the table, the baseline model MobileNetV3 achieved a classification accuracy of 98.45% on the PatternNet dataset. The introduction of the various modules led to significant performance improvements; notably, the simultaneous integration of all three modules pushed accuracy to approximately 99.72% with minor fluctuations—a 1.27% increase over the baseline—while also achieving optimal results in terms of inference time and FPS. This not only validates the soundness and effectiveness of the proposed framework but also confirms the suitability of the Pattern dataset as a high-quality benchmark for scene classification.

Firstly, the introduction of the MSFE module alone resulted in an accuracy increase of approximately 1.1%. Although the gain in this single metric was modest, the module achieved inference times and FPS rates second only to the full model while maintaining the lowest CPU memory footprint; its unique ability to capture multi-scale features makes it particularly well-suited for edge detection. In comparison, the DFWF module achieved a performance boost of about 1.17% while maintaining low GPU memory usage and computational complexity, demonstrating that dynamic channel weight allocation effectively suppresses redundant information. The introduction of the hierarchical CBAM module alone yielded the highest individual gain approximately 1.23%—highlighting how the combination of channel and spatial attention effectively suppresses background noise and enhances responses in key regions. Regarding module combinations, the MSFE + DFWF pairing achieved an accuracy of 99.69%; the subsequent addition of the hierarchical CBAM raised the accuracy to 99.72% and reduced the standard deviation from ±0.073 to ±0.014, demonstrating that the hierarchical CBAM significantly enhances training stability and model robustness.

In summary, while MSFE and DFWF form the core of multi-scale representation and dynamic weighting, the hierarchical CBAM serves as a lightweight yet effective refinement module that boosts overall robustness, stability, and feature discriminative capability with negligible additional cost. The synergistic integration of these three modules significantly enhances the model’s classification performance in complex scenarios.

### 5.3. The Impact of Data Augmentation

During the training process, data augmentation was applied to the input images through random rotation, horizontal and vertical translation, shearing, zooming, and horizontal flipping. This strategy generates a more diverse set of training samples than the original images. To evaluate the effectiveness of data augmentation, comparative experiments were conducted on all six datasets with and without augmentation, as summarized in Table 6 below. Experimental results show that the application of data augmentation significantly enhances the data on five different datasets, particularly the WHU-RS19, AID, and CLRS datasets, with an increase in accuracy of approximately 10%. The results demonstrate that data augmentation effectively increases sample diversity and enhances the model’s robustness against variations in object orientation, scale, and spatial distribution. However, the effect of data augmentation was not pronounced for the Pattern dataset; this may be attributed to its inherently high data quality and well-defined scene characteristics, making it suitable for model detection in remote sensing scene classification tasks. Nevertheless, the overall results confirm that data augmentation serves as an effective regularization strategy and contributes significantly to the generalization capability of the proposed framework.

### 5.4. Analysis of Experimental Results

To further evaluate the effectiveness, robustness, and practical applicability of the proposed model, we conducted multiple independent experiments across six benchmark remote sensing scene classification datasets. We recorded the mean accuracy and standard deviation (MA ± Std) to assess statistical stability. Additionally, we tracked the best stable classification accuracy, inference time, FPS, CPU memory usage, and peak GPU memory consumption to comprehensively evaluate the model’s efficiency for lightweight deployment.

As shown in Table 7, MDC-MobileNetV3 demonstrated excellent classification performance across all six benchmark datasets. Specifically, classification accuracies on the UC Merced, WHU-RS19, NWPU-Resisc45, AID, CLRS, and Pattern datasets reached 99.52%, 91.54%, 96.48%, 92.43%, and 99.72%, respectively. Notably, the standard deviation on the Pattern dataset was only 0.014, indicating that the model maintained highly consistent convergence across multiple validation runs. The results for the Pattern and UC Merced datasets suggest that the relatively clear scene boundaries and well-structured categories in these datasets allowed the model to effectively leverage multi-scale feature extraction and attention mechanisms to achieve high-precision classification.

Although the UC Merced and WHU-RS19 datasets contain relatively few samples, the model performed impressively; notably, it achieved a mean accuracy of 99.285 ± 0.284% on the UC Merced dataset, fully demonstrating its robust performance under data-limited conditions. In contrast, the standard deviation for WHU-RS19 was 1.381%; this variation may be attributed to the dataset’s small size and significant intra-class diversity, which made it challenging to maintain consistent stability across experiments, yet the results still confirm the model’s effectiveness in scenarios with limited data. In addition to classification performance, deployment efficiency is a key criterion for lightweight remote sensing scene classification. As shown in Table 7, the proposed method achieves a single-image inference time of 145–174 ms across different datasets while maintaining a frame rate of approximately 5.75–6.86 FPS. Furthermore, CPU memory usage remains around 3.2 GB, and peak GPU memory consumption stabilizes at 349 MB after deployment. These results demonstrate that the model maintains a lightweight profile regarding computational overhead while exhibiting excellent deployment capabilities.

Overall, the experimental results indicate that MDC-MobileNetV3 achieves high classification accuracy alongside outstanding statistical stability and deployment efficiency, striking a favorable balance among classification performance, lightweight design, and robustness.

**Figure 15:**
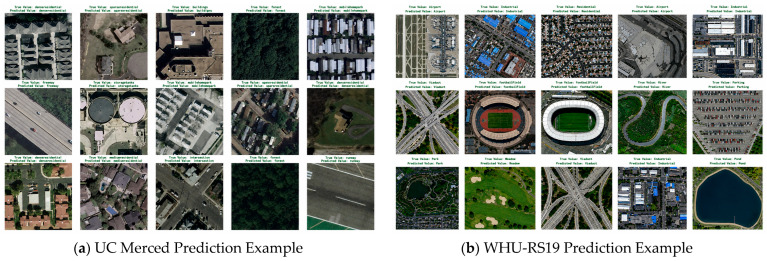

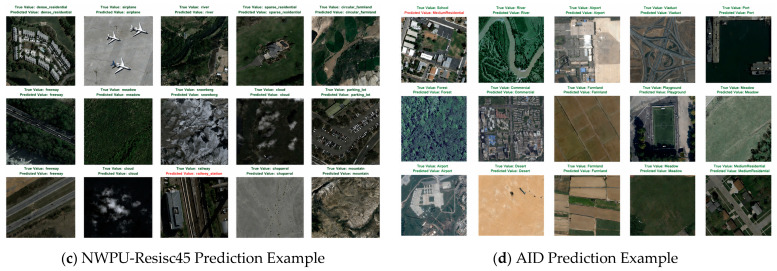

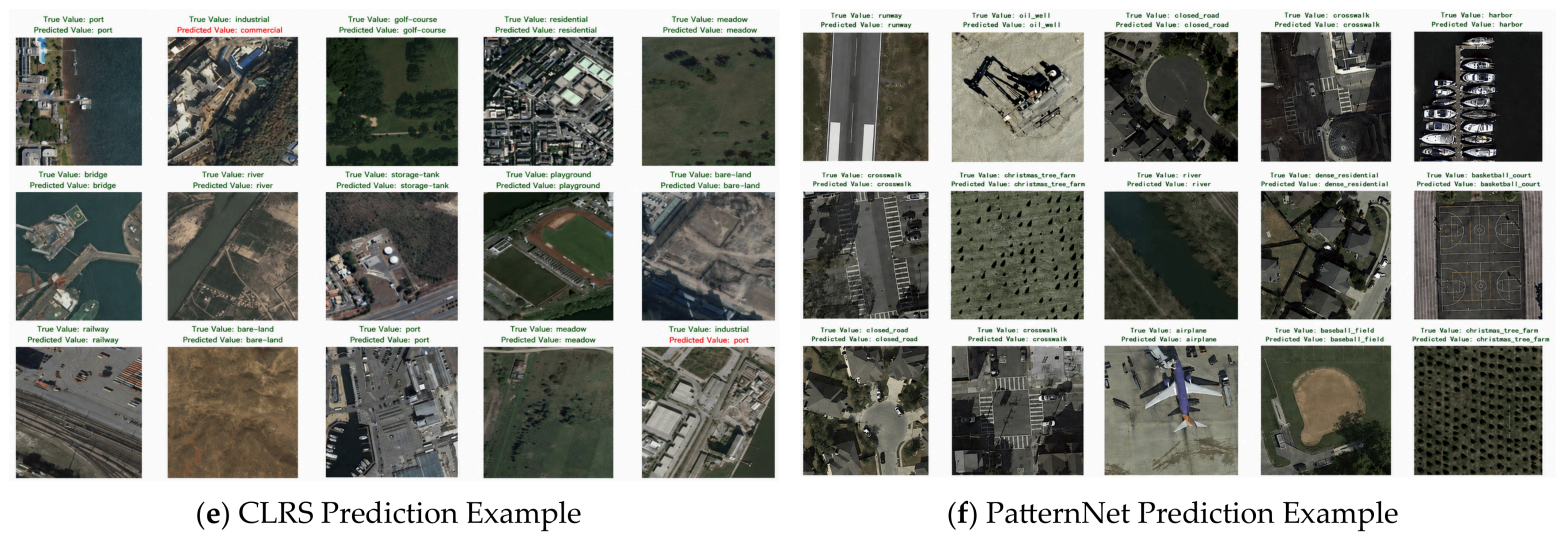
Visualization of Classification Results for Each Dataset.

Figure 15 shows the results of some predicted instances from the model across six datasets. The visualization demonstrates that the model is capable of accurately identifying remote sensing targets across different scales and within complex backgrounds. In most cases, the network successfully recognizes diverse land-cover features, including airports, viaducts, and harbors, with predicted labels exhibiting high consistency with the corresponding ground-truth categories. These observations provide intuitive evidence of the strong spatial semantic representation capability of the proposed framework.

On the UC Merced dataset, the model accurately distinguishes categories with significant structural differences, such as DenseResidential, Forest, StorageTank, and Intersection, indicating that the integration of the backbone network with the proposed MSFE module provides effective spatial structure perception capability. In the WHU-RS19 and AID datasets, the model also maintains a high recognition rate for categories with complex textures and spatial layout features, such as Viaduct, River, Playground, Pond, and Airport. This shows that the Hierarchical CBAM module effectively enhances the response to key areas and reduces background noise interference. However, for some categories with highly similar textural structures, the model still exhibits a small number of misclassifications.

Furthermore, by examining the few instances of prediction errors shown in the Figure 15 (marked in red), it is evident that misclassifications primarily occur between scenes with high spatial correlation. For example, in the NWPU Resisc45 dataset, ‘Railway’ is incorrectly predicted as ‘Railway Station’ and ‘School’ is misclassified as ‘MediumResidential’ in the AID dataset, while in the CLRS dataset, ‘Industrial’ is misclassified as ‘Port.’ This is primarily due to the presence of overlapping visual elements in the scene images, which exhibit similar land cover texture distributions or spatial arrangement patterns. However, overall, the model’s predictions remain highly consistent and demonstrate strong feature discrimination capabilities.

### 5.5. Visual Analytics

**Figure 16:**
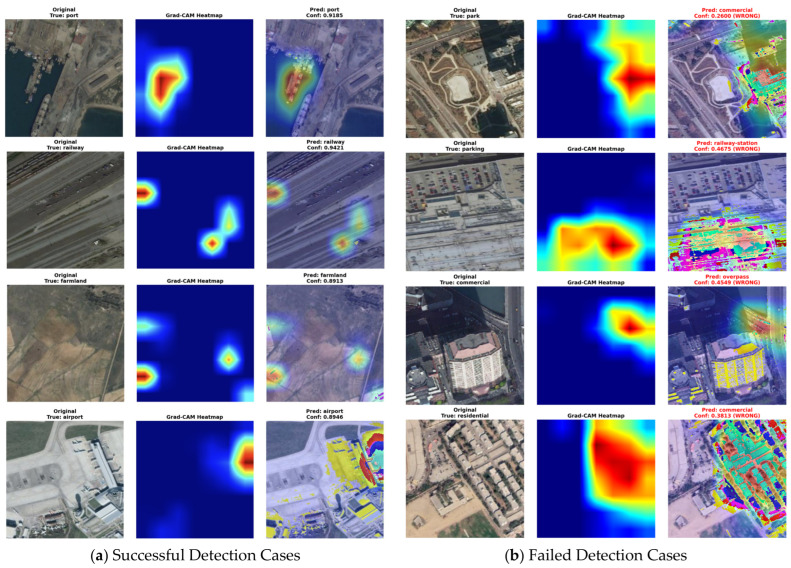
Grad-CAM Thermal Visualization.

To further verify whether the proposed model framework effectively learns key discriminative regions, this paper conducted Grad-CAM visualization analysis on correctly and incorrectly classified samples from the CLRS and Pattern datasets; representative cases are shown in Figure 16a and Figure 16b, respectively. By considering both scenarios, we investigated the model’s decision-making capabilities from the perspectives of feature localization and semantic discrimination.

As illustrated in Figure 16a, the model’s high-response regions are primarily concentrated on key structural elements of the target and areas containing semantic information. In the Port scene category, the heatmaps reveal that the proposed framework accurately focuses on: vessel berthing areas, harbor boundary structures, and land–water transition regions, while effectively suppressing large-scale background water-body interference. This observation indicates that the model has successfully learned the discriminative characteristics associated with harbor-related land-cover structures. For the Railway scene category, the model primarily concentrates on railway track arrangements and track intersection structures, demonstrating that the spatial attention mechanism effectively enhances the representation capability of linear structural features. In the Airport scene category, the high-response regions are mainly distributed around aprons, terminal buildings, runway boundaries, and aircraft-concentrated areas, while the influence of background interference, such as surrounding road regions, is substantially suppressed. This further demonstrates that the model is not only capable of perceiving local objects but can also perform classification judgements by integrating global layout structures.

In contrast, the failure case shown in Figure 16b reveals the method’s limitations regarding scene overlap and high spatial feature similarity between scenes; for instance, in the case where ‘Park’ was misclassified as ‘Commercial’, the heat map indicates that the model focused on surrounding transportation facilities and structures while overlooking vegetation-related textural regions, as the presence of roads, parking lots, and buildings created visual characteristics typical of ‘Commercial’, thereby leading to ambiguity in semantic interpretation. In the case which ‘Parking’ was misclassified as ‘Railway Station’, the model exhibited a strong response to the slender, linear structures in the lower part of the image, leading it to associate the scene with a railway; additionally, the presence of numerous densely packed vehicles made it easy to confuse the scene with ‘Railway Station’. Furthermore, among the samples ‘Commercial’ was misclassified as ‘Overpass’, elevated road infrastructure dominated the distribution of attention and suppressed the response to building complexes. Meanwhile, in cases which ‘Residential’ was misclassified as ‘Commercial’, the densely arranged building clusters and road networks exhibited spatial characteristics highly similar to ‘Commercial’ environments, thereby leading to the misclassification. The above phenomena indicate that, during feature aggregation, dominant geometric structures tend to obscure contextual semantic information, while also making it difficult to correctly distinguish between highly similar scene categories.

Overall, the framework proposed in this paper effectively suppresses irrelevant background noise and automatically focuses on key structural regions to achieve accurate classification in most scenarios. However, prediction errors may still occur in situations involving significant scene overlap and complex urban structures. Grad-CAM visualizations reveal that the model tends to focus on regions containing high-semantic-value features rather than random background elements; consequently, errors are primarily attributed to the extreme similarity between categories. This finding underscores the necessity of incorporating mechanisms for robust global contextual modeling and fine-grained semantic discrimination in future work.

Based on the experimental results and visualization analysis above, the lightweight multi-module collaborative attention framework proposed in this paper provides an efficient and interpretable approach for remote sensing scene classification. The competitive performance across six benchmark datasets, particularly the favorable balance between high classification accuracy and low parameter complexity, validates the effectiveness of the MSFE–DFWF–CBAM design. Furthermore, compared with traditional single-scale CNNs, the model proposed in this paper is better equipped to address challenges in remote sensing scene classification, such as high inter-class similarity, significant intra-class variation, complex background noise, and substantial variations in object scale. Additionally, compared with Transformer-type models, the proposed method achieves high classification performance while maintaining low parameter counts and computational complexity, making it particularly suitable for edge devices and lightweight remote sensing intelligent interpretation scenarios. Overall, the experimental results fully validate the effectiveness, robustness, and interpretability of the proposed method in remote sensing scene classification tasks.

### 5.6. Discussion with Recent Lightweight Methods

In this subsection, we compare our method with recently published lightweight hybrid feature attention networks and investigate the impact of multi-scale information, attention mechanisms, and feature interactions on the model. It is important to note that the comparative models were evaluated under significantly different conditions—including variations in training rates, training epochs, optimization strategies, and pre-training schemes. Consequently, a direct quantitative comparison of reported classification accuracies would not constitute a rigorous assessment. Therefore, the discussion here focuses on methodological differences rather than a comparison of specific numerical performance metrics. Specific details of the model results are shown in Table 8 below.

Briefly considering the table above, MS^2^AP [32] integrates multi-scale dilated convolutions and a residual channel-spatial attention mechanism into the VGG16 backbone, achieving the best results among the listed models: 99.09% on UC Merced (50%), 96.89% on AID (50%), and 92.27% on NWPU-Resisc45 (10%). Newer lightweight designs—such as AEBANet [59] and HLAE-Net [60]—utilize MobileNetV2 and ResNet50 as backbones, respectively, while incorporating techniques like multi-layer feature fusion, adaptive branch attention, hierarchical feature collaborative extraction, and dual-coordinate spatial attention mechanisms. These models demonstrate excellent performance across various training ratios, achieving high classification accuracies of 95.52% on AID (20%), 99.67% on UC Merced (80%), and 94.01% on NWPU-Resisc45 (20%). MCFFCNet [61] employs multi-CNN feature fusion and an efficient backbone but lacks a dedicated attention mechanism, relying instead on post-fusion feature selection; this limitation may hinder its ability to suppress complex background interference in high-resolution aerial imagery.

In contrast, the MDC-MobileNetV3 proposed in this paper adopts MobileNetV3 as its backbone and introduces a collaborative processing pipeline comprising a parallel Multi-Scale Feature Extraction (MSFE) module, a Dynamic Feature Weighting Fusion (DFWF) mechanism, and a hierarchical CBAM attention module. As shown in the table, at an 80% training ratio, the model achieves 99.72% and 99.52% accuracy on the Pattern and UC Merced datasets, respectively, while maintaining highly competitive classification performance on more challenging datasets such as AID (97.35%) and NWPU-Resisc45 (96.48%).

Overall, compared to recent lightweight remote sensing scene classification frameworks, the proposed MDC-MobileNetV3 demonstrates the effectiveness of integrating multi-scale feature extraction, dynamic feature fusion, and hierarchical attention mechanisms within a unified, lightweight architecture. It is still capable of achieving state-of-the-art classification accuracy with extremely low computational overhead, thereby offering a practical balance between classification performance and deployment efficiency for remote sensing scene interpretation tasks.

## 6. Conclusions

This paper presented MDC-MobileNetV3, a lightweight remote sensing scene classification framework designed to address the challenges posed by large-scale object variability, complex background interference, and semantic ambiguity among scene categories. The proposed architecture integrates three complementary components, namely Multi-Scale Feature Extraction (MSFE), Dynamic Feature Weighted Fusion (DFWF), and a hierarchical CBAM-based attention mechanism, enabling multi-scale semantic information enhancement and adaptive feature recalibration, significantly improving the model’s feature representation ability and classification performance while maintaining computational efficiency.

Experimental validation was conducted on six benchmark datasets—UC Merced, WHU-RS19, NWPU-Resisc45, AID, CLRS, and PatternNet. The results demonstrate that the proposed model exhibits superior performance and robust generalization capabilities across scenarios of varying complexity; it achieves state-of-the-art classification accuracy (reaching 99.72% and 99.52% on the PatternNet and UC Merced datasets, respectively) while maintaining a compact architecture with only 4.35M parameters and low computational complexity of 0.57 GFLOPs. Additional analyses based on confusion matrices, prediction visualizations, and Grad-CAM activation maps further revealed that the model learns semantically meaningful and spatially discriminative representations. Our method effectively focuses on key texture discrimination regions, such as runways, railway networks, port facilities, and agricultural patterns, significantly enhancing the model’s semantic awareness and feature interpretability in target regions.

Future research will explore hybrid CNN–Transformer architectures and cross-scale global dependency learning to further strengthen global contextual relationships and fine-grained scene understanding in large-scale remote sensing applications.

## Figures and Tables

**Table 1:** Basic information of the six datasets.

Dataset	Classes	Samples Per Class	Image Size	Number
UC Merced	21	100	256 × 256	2100
WHU-RS19	19	50–55	600 × 600	1005
AID	30	240–420	600 × 600	10,000
NWPU-Resisc45	45	700	256 × 256	31,500
CLRS	25	600	256 × 256	15,000
PatternNet	38	800	256 × 256	30,400

**Table 2:** Experimental Results of Comparison between SOTA Models on AID.

Model	Accuracy	Params (M)	FLOPs (G)
VIT-B16	88.85%	85.825	**35.228**
EfficientNetB0	91.50%	4.093	0.787
EfficientNetB1	90.15%	6.619	1.400
EfficientNetB2	90.60%	7.816	2.021
EfficientNetB3	89.75%	10.836	1.956
ConvNext	85.95%	20.627	6.142
ShuffleNetV2	88.35%	1.302	**0.289**
NASNet	81.55%	4.306	1.135
MobileNetV2	87.30%	2.302	0.599
MobileNetV3	91.55%	3.025	0.433
MobileNetV4	84.60%	**1.016**	1.393
**MDC-MobileNetV3 (ours)**	**97.35%**	4.350	0.570

**Table 3:** Experimental Results Comparing Traditional Models on AID.

Model	Accuracy	Params (M)	FLOPs (G)
ResNet50	92.25%	23.657	7.724
ResNet50V2	89.40%	23.634	6.970
VGG16	86.95%	23.169	2.524
VGG19	84.05%	28.479	3.204
DenseNet121	92.95%	7.068	5.669
**MDC-MobileNetV3 (ours)**	**97.35%**	**4.350**	**0.570**

**Table 4:** MDC-MobileNetV3 AID Classification Performance Report.

Class	Precision	Recall	F1 Score	Support	Class	Precision	Recall	F1 Score	Support
Airport	0.967	1.000	0.983	58.0	Mountain	1.000	0.986	0.993	73.0
BareLand	1.000	0.902	0.948	61.0	Park	0.942	0.929	0.935	70.0
BaseballField	1.000	1.000	1.000	46.0	Parking	0.988	0.988	0.988	85.0
Beach	1.000	1.000	1.000	89.0	Playground	0.946	0.972	0.959	72.0
Bridge	0.973	0.948	0.961	77.0	Pond	1.000	0.976	0.988	82.0
Center	0.913	0.875	0.894	48.0	Port	0.986	1.000	0.993	70.0
Church	0.875	0.933	0.903	45.0	RailwayStation	0.961	0.942	0.951	52.0
Commercial	0.907	1.000	0.951	78.0	Resort	0.857	0.889	0.873	54.0
DenseResidential	1.000	0.975	0.987	80.0	River	1.000	1.000	1.000	81.0
Desert	0.932	1.000	0.965	55.0	School	0.944	0.893	0.918	75.0
Farmland	0.977	0.988	0.982	85.0	SparseResidential	0.954	1.000	0.976	62.0
Forest	1.000	1.000	1.000	50.0	Square	0.945	0.852	0.897	61.0
Industrial	0.974	0.925	0.949	80.0	Stadium	0.963	0.963	0.963	54.0
Meadow	1.000	0.982	0.991	56.0	StorageTanks	0.969	0.969	0.969	65.0

**Table 5:** Model Ablation Experiment Analysis.

Model	MA ± Std (%)	Params (M)	FLOPs (G)	Infer-Time (ms)	FPS	CPU	GPU
MobileNetV3	98.450 ± 0.028	**3.0329**	**0.4327**	160.576	6.228	3001.500	**48.090**
MSFE	99.548 ± 0.046	4.3020	0.5701	155.254	6.441	**2147.406**	348.760
DFWF	99.620 ± 0.059	3.7180	0.4341	164.403	6.083	3073.641	83.379
CBAM	99.681 ± 0.023	3.4876	0.4342	156.322	6.397	3012.859	81.839
MSFE + DFWF	99.691 ± 0.073	4.3351	0.5702	165.021	6.060	3277.309	348.710
MSFE + CBAM	99.663 ± 0.022	4.3188	0.5703	165.286	6.050	3376.496	348.207
DFWF + CBAM	99.706 ± 0.039	3.9496	0.4352	156.325	6.397	3262.977	86.280
MSFE + DFWF + CBAM	**99.713 ± 0.014**	4.3518	0.5704	**155.116**	**6.447**	3293.883	348.679

**Table 6:** Data Augmentation Comparison Table.

Dataset	Data Augmentation	No-Data Augmentation
UC Merced	99.52%	98.57%
WHU-RS19	91.54%	80.10%
NWPU-Resisc45	96.48%	95.86%
AID	97.35%	85.60%
CLRS	92.43%	72.17%
PatternNet	99.72%	99.72%

**Table 7:** Classification Accuracy, Statistical Stability, and Deployment Performance of the Model on Six Benchmark Datasets.

Dataset	Accuracy	Loss	MA ± Std (%)	Infer-Time (ms)	FPS	CPU	GPU
UC Merced	99.52%	0.617	99.285 ± 0.284	168.168	5.946	3291.203	348.806
WHU-RS19	91.54%	0.968	88.557 ± 1.381	145.679	6.864	2828.871	348.812
NWPU-Resisc45	96.48%	0.814	96.384 ± 0.117	159.202	6.281	3291.410	348.571
AID	97.35%	0.729	97.170 ± 0.228	163.982	6.098	3275.441	348.809
CLRS	92.43%	0.864	92.135 ± 0.221	173.891	5.751	3286.953	348.712
PatternNet	99.72%	0.686	99.713 ± 0.014	155.116	6.447	3293.883	348.679

**Table 8:** Comparison of Recent Advanced Lightweight Hybrid Multi-Scale Attention Methods for Remote Sensing Scene Classification.

Method	Backbone	Multi-Scale	Attention	Lightweight	Performance on Key Datasets
MSA-Network (2021) [31]	ResNet	Multiscale Module	Channel Attention /Position Attention	×	UC Merced(80%)-98.96% UC Merced(50%)-97.80% AID(50%)-96.01% AID(20%)-93.53% NWPU-Resisc45(20%)-93.52% NWPU-Resisc45(10%)-90.38%
MS^2^AP (2021) [32]	VGG16	Multi-scale dilated convolutional operator	Residual Channel-Spatial Attention	√	UC Merced(80%)-99.45% **UC Merced(50%)-99.09%** **AID(50%)-96.89%** AID(20%)-95.42% NWPU-Resisc45(20%)-93.91% **NWPU-Resisc45(10%)-92.27%**
AEBANet (2025) [59]	MobileNetV2	Multi-Level Feature Fusion	Adaptive Enhanced Branch Attention	√	UC Merced(80%)-99.52% UC Merced(50%)-98.48% AID(50%)-96.67% **AID(20%)-95.52%** NWPU-Resisc45(20%)-93.12% NWPU-Resisc45(10%)-91.10%
HLAE-Net (2025) [60]	ResNet50	Hierarchical Feature Collaborative Extraction	Dual Coordinate Spatial Attention/Multiscale Spatial/ Channel Attention	√	**UC Merced(80%)-99.77%** UC Merced(50%)-98.76% AID(50%)-96.50% AID(20%)-94.26% RSSCN7(80%)-97.14% **NWPU-Resisc45(20%)-94.01%** NWPU-Resisc45(10%)-91.88%
MCCFFCNet (2026) [61]	ResNet50/ MobileNetV2/ DenseNet121/ EfficientNetB0	Multi-CNN	×	√	**EuroSAT(80%)-96.28%** NWPU-Resisc45(80%)-94.52%
MDC-MobileNetV3 (ours)	MobileNetV3	Multi-Scale Feature Extraction	Dynamic Feature Weighted Fusion/ hierarchical CBAM	√	UC Merced(80%)-99.52% WHU-RS19(80%)-91.54% **NWPU-Resisc45(80%)-96.48%** AID(80%)-97.35% **CLRS(80%)-92.43%** **PatternNet(80%)-99.72%**

## Data Availability

The data may be requested from the author if required. This is because the relevant data is currently being used in ongoing research.
